# Clinical Efficacy and Safety Profile of Caplacizumab for Acquired Thrombotic Thrombocytopenic Purpura

**DOI:** 10.7759/cureus.5263

**Published:** 2019-07-29

**Authors:** Muhammad Haisum Maqsood, Kinza Rubab, Muhammad Zaigham Maqsood

**Affiliations:** 1 Internal Medicine, King Edward Medical University / Mayo Hospital, Lahore, PAK; 2 Surgery, District Headquarter Hospital, Faisalabad, PAK

**Keywords:** caplacizumab, acquired thrombotic thrombocytopenic purpura, ttp

## Abstract

Thrombotic thrombocytopenic purpura (TTP) is usually defined as microangiopathy characterized by low platelet count and low red blood cell count, i.e., hemolytic anemia. It can either be acquired or immune-mediated. TTP requires quick diagnostic identification and emergent management. According to the evidence-based guidelines, the recommended therapy is plasma exchange and immunosuppression. Caplacizumab is used alongside the standard recommended therapy. Caplacizumab is a monoclonal antibody (Mab) that binds to von Willebrand factor (VWF). This prevents A1 VWF to bind platelet glycoprotein 1b receptor. The recommended dosage for this drug is 10mg. At the start, 10mg intravenous (IV) dose is given before plasma exchange, followed by daily 10mg subcutaneous (SC) dose after plasma exchange. Moreover, the SC dose is continued even after the daily plasma exchange is stopped. This review aims to consolidate findings related to the efficacy of this recently approved drug.

## Introduction and background

The condition

Thrombotic thrombocytopenic purpura (TTP) is usually defined as microangiopathy characterized by low platelet count and low red blood cell count, i.e., hemolytic anemia. It can either be acquired or immune-mediated [1-4]. According to an estimate, the incidence of TTP is 2-4 cases per million per annum [5]. It is more common in females than in males (3:2 ratio) and the median age for this disease is in the fourth decade [6]. In 1982, it was hypothesized by Moake and his coworkers that ultra-large von Willebrand factor (VWF) leads to platelet aggregation in the microvasculature [7]. Autoantibodies inhibit the activity of the VWF-cleaving-protease ADAMTS13, and this leads to activation of platelets resulting in microvascular thrombosis [8-11]. This microvascular thrombosis is responsible for multi-organ ischemia, ultimately leading to life-threatening complications. There might be cardiac, neurologic or renal injury depending upon the organ affected by tissue ischemia [12-17]. The prominent and consistent features of TTP are hemolytic anemia (red blood cell breakdown) and thrombocytopenia (low platelet count). Central nervous system and gastrointestinal manifestations are also occasionally present with mild renal involvement [18]. TTP is a disease that is still under-diagnosed and is a diagnostic and therapeutic challenge. The diagnosis is mainly based on history, physical examination and laboratory workup, and can be confirmed by ADAMTS13 level (less than 10%). This rare autoimmune disease leads invariably to a fatal outcome if treatment is not started [19-21].

The treatment

TTP requires quick diagnostic identification and emergent management, as it is a medical emergency. According to the evidence-based guidelines, the recommended therapy is plasma exchange and immunosuppression [22]. The target for initial treatment is to stop thrombosis at the earliest possible time. The post-thrombotic process is associated with high morbidity and mortality [23-24]. The use of therapeutic plasma exchange (TPE) was the first advancement in the treatment of this disease. Despite this, the mortality among patients remained 10 to 20% [25]. In 1998, the revelation of ADAMTS13 provided a rationale for B-cell depleting therapeutic strategies [24]. The addition of anti-CD20 (cluster of differentiation 20) monoclonal antibody, rituximab, to the standard regimen was the next achievement in managing the disease, i.e., immunosuppressive management [26]. The UK and French reports concluded that rituximab resulted in short hospital stays, longer remission and fewer relapses [27-28]. As TTP is an autoimmune disease, the indication to use corticosteroid has limited evidence [29-32].

Caplacizumab

Caplacizumab is a monoclonal antibody (Mab) that binds to von Willebrand factor (VWF). This prevents A1 VWF to bind platelet glycoprotein 1b receptor [33-35]. Caplacizumab was globally approved on September 3, 2018, for the treatment of adults with TTP concurrently with plasma exchange and immunosuppression [36-37]. At the start, 10mg intravenous (IV) dose is given before plasma exchange followed by a daily 10mg subcutaneous (SC) dose after plasma exchange. Thereafter, the daily SC dose is continued, at the same quantity, after the daily plasma exchange is stopped [36]. Maximum concentration (Cmax) of caplacizumab is reached in 6-7 hrs. This drug is almost completely absorbed and enters systemic circulation rapidly after SC administration, and its volume of distribution (Vd) is 6.33 L in the TTP patients [36]. It has been reported that caplacizumab is used until the ADAMTS13 levels returned to normal. It also stops microthrombi proliferation and the consequence of the thrombosis, i.e., occlusion and ischemic injury, especially to the end organs until rituximab and corticosteroids are effective [38].

This review article aims to consolidate the literature review regarding the efficacy of caplacizumab, a newly approved drug for TTP.

## Review

Efficacy of caplacizumab treatment

Time for Platelet Count Restoration

The median time to thrombocytopenia rectification is significantly shorter with caplacizumab as compared to the placebo (2.69 versus 2.88 days), and patients who are treated with caplacizumab have one-and-half times greater chance of platelet counts returning to normal [21]. The study by Peyvandi et al. has also revealed that caplacizumab is associated with quick resolution of thrombocytopenia [39]. It has been reported that caplacizumab results in 39% faster normalization of platelet counts (p=0.005) [39-41].

Thrombotic Thrombocytopenia Recurrence

It has been reported in the literature that recurrence of TTP is significantly lower with caplacizumab than with placebo (12% versus 38%) [21].

Refractory Disease

The refractory disease can be defined in two ways: either no platelet response after seven days, despite daily plasma-exchange therapy (6% in caplacizumab group vs 22% in placebo) or failure of increase in platelet count two times from baseline after four days of standard treatment (zero vs 11%) [39]. Refractoriness is a poor prognostic factor for TTP. It has been shown that 11% of patients in the placebo group developed refractoriness and zero percent of patients developed refractoriness in Caplacizumab group (p=0.06) [21]. Caplacizumbab resulted in a reduction in the patients’ refractoriness to treatment [42].

Relapse

A relapse is an acute flare of TTP occurring after one month of plasma exchange therapy [40]. It has been reported in a study that 11 patients (out of 35) in the caplacizumab group, as compared to three (out of 37) in the placebo group, had a relapse during the follow-up [41]. This study is backed by another study showed that eight (out of 35) patients who received caplacizumab and zero patient who received placebo group relapsed within thirty days [40].

Complete Remission

It has been demonstrated that caplacizumab use is most likely associated with complete remission (i.e. platelet count normalization without any exacerbation) rate than the placebo [39,43].

Exacerbation

Exacerbation is a recurrence of thrombocytopenia which occurs during plasma exchange or within one month of plasma exchange [40]. The literature revealed that caplacizumab resulted in fewer exacerbations during the treatment period as compared to the placebo: three patients (out of 35) for caplacizumab vs. 11 (out of 37) for placebo [39].

Plasma Exchange

Plasma exchange was introduced in the late 1970s and it was shown to reduce mortality from greater than 90% to about 20% [40]. The literature showed that caplacizumab treatment group required fewer plasma exchange sessions and had short hospital stays than those who are in the placebo treatment group (7.7 days versus 11.7 days for placebo) [21,39]. An analysis reported that the number of days plasma was exchanged and the volume of plasma exchanged were lower in the caplacizumab group [42].

Adverse Effects

It has been revealed that the most common adverse event of caplacizumab was bleeding from mucosal and cutaneous surfaces (more common with caplacizumab (65%) versus placebo (48%)) [21]. Other reactions with caplacizumab treatment included fatigue, urticaria, injection site reaction, fever, myalgia, cerebral infarction, and dyspnea [43]. The HERCULES trial (A Safety and Effectiveness Study of the Herculink Elite Renal Stent to Treat Renal Artery Stenosis) showed that the frequency of patients experiencing treatment-emergent adverse effects was significant in caplacizumab as compared to placebo (57.8% versus 43.8%). The frequency of drug discontinuation was higher with placebo with respect to caplacizumab [21].

Risk of Bleeding

Caplacizumab alters the function of von Willebrand factor and indirectly interferes with the physiologic mechanism of hemostasis [21]. Hence, bleeding-related events were significantly higher with caplacizumab as compared to placebo (54% versus 38%), but they were mild enough that the need for any intervention was not necessary [40]. According to a study, out of 101 bleeding-related adverse events, only 3% were severe enough to qualify for receiving an intervention [42].

Thromboembolic Events

According to an analysis, four thromboembolic events were reported in four patients in the caplacizumab group, while 20 such events were reported in 14 patients in the placebo group [39]. This analysis shows that caplacizumab has the potential to reduce the risk of thromboembolic events associated with TTP [39].

Long-Term Complications

TTP is associated with an increased risk for other autoimmune diseases as well. Major thromboembolic events can result in cognitive and physical disabilities [39]. This necessitates the need for proper recognition and management [6].

Markers of Organ Damage

After treatment, second-day lactate dehydrogenase (LDH) was higher in the caplacizumab than in placebo. However, the time to normalize LDH was three days in caplacizumab and four days in placebo [6,42]. Creatinine normalization takes four days with caplacizumab intervention versus six days with placebo intervention [6,42].

Effect on von Willebrand factor-ristocetin cofactor

The literature showed that caplacizumab intervention resulted in a fast decline in VWF-ristocetin cofactor activity to <20%. VWF antigen and factor VIII also showed a temporary decline. This is due to increased clearance of the caplacizumab-von Willebrand factor complex as compared to the unbound von Willebrand factor [21,42].

Table 1 summarizes the important characteristics of caplacizumab treatment for acquired TTP.

**Table 1:** Highlights of the main features of this review. TTP: Thrombotic thrombocytopenic Purpura.

Main Features	Findings	References
Time for Platelet Count Restoration	Thrombocytopenia rectification is faster with caplacizumab as compared to the placebo (2.69 versus 2.88 days), and patients who are treated with caplacizumab have one-and-half times greater chance of platelet counts returning to normal.	[21,39]
Thrombotic thrombocytopenia recurrence	Recurrence of TTP is significantly lower with caplacizumab than with placebo.	[21]
Refractory Disease	There are fewer chances of refractoriness with caplacizumab: 11% of patients in the placebo group developed refractoriness and zero percent of patients developed refractoriness in the caplacizumab group. Caplacizumbab also results in a reduction in the patients’ refractoriness to treatment.	[21,42]
Relapse	Caplacizumab is associated with a higher relapse rate as compared to placebo. A study reported that 11 patients (out of 35) in the caplacizumab group, as compared to three (out of 37) in the placebo group, had a relapse during the follow-up. This study is backed by another study showed that eight (out of 35) patients who received caplacizumab and zero patient who received placebo group relapsed within thirty days.	[40,41]
Complete Remission	Caplcizumab use is most likely associated with complete remission (i.e., platelet count normalization without any exacerbation) rate than the placebo.	[39,43]
Exacerbation	Caplacizumab results in fewer exacerbations during the treatment period as compared to the placebo: three patients (out of 35) for caplacizumab vs. 11 (out of 37) for placebo.	[39]
Plasma Exchange	The literature shows that caplacizumab treatment group required fewer plasma exchange sessions and had shorter hospital stays than those who are in the placebo treatment group (7.7 days versus 11.7 days for placebo). An analysis reported that the number of days plasma exchange was done and the volume of plasma exchange were lower in the caplacizumab group.	[21,39,42]

Future research

There is an ongoing clinical trial for caplacizumab, a prospective follow-up Post-HERCULES study to evaluate the long-term safety and efficacy of caplacizumab [21,39,44]. The future research must focus on potent, long-lasting and selective immunomodulatory therapeutic strategies with the capability of removing or neutralizing autoantibodies towards ADAMTS13. Administration of recombinant ADAMTS13 can be administered for cases of TTP [40].

Discussion

Thrombotic thrombocytopenic purpura is a rare hematologic disorder that is characterized by thrombocytopenia and purpura [1-4]. It is surprising that the mechanism of the disease is widely understood but the mortality rate is as high as 20% [22,43].

The current treatments options do not correct the microvascular thrombosis [21]. Thus, our review focused on the targeted therapeutic strategy, caplacizumab. Caplacizumab is a humanized monoclonal antibody which interferes with the adhesion of von Willebrand factor multimers with platelets, which is essential for thrombus formation [33-35]. Our review included seven articles and involves a comprehensive assessment of the beneficial and adverse effects of this drug. The results of our literature review suggest that caplacizumab helps in normalizing the platelet counts at a faster rate and to a greater extent as compared to the placebo [39-42]. Patients of TTP on caplacizumab need less plasma exchange to show an improved outcome [21,39,40,42]. It has also been demonstrated that this therapeutic drug leads to fewer exacerbations as compared to the placebo [39,40]. Most importantly, caplacizumab is associated with a high complete remission rate and a low recurrence of this autoimmune disorder [21,39,42]. It has also been revealed that patients on caplacizumab are less likely to develop refractory disease [40,41]. However, the relapse rate is reported to be higher amongst the patients on caplacizumab as compared to the placebo [41]. It is evident from this review that caplacizumab is associated with some adverse effects as well. It has been reported in the literature that episodes of mucocutaneous bleed occur at a higher rate as compared to the placebo (54% vs 38%) [40,42]. This can be explained by caplacizumab interfering with the von Willebrand factor. However, the percentage of patients experiencing thromboembolic events was lower with caplacizumab as compared to the placebo (11.4% vs 43.2%) [39]. Though less in number, such thromboembolic events might lead to long-term cognitive and physical disabilities in patients experiencing TTP [6,39].

Though our review involves a comprehensive analysis of many of the aspects of this emerging therapeutic option, our study should be interpreted in the light of potential limitations. Firstly, the lack of evidence and trials lead to incomplete evaluation of the effectiveness of this drug. Secondly, all the studies that were included in our review involved the comparison with placebo; whereas a placebo is not available in clinical practice. Thus we believe that decisions in clinical practice should rely mainly on direct comparisons of active treatments available to clinicians. Thirdly, we might not have explored and evaluated all the parameters of this monoclonal antibody.

## Conclusions

It can be concluded that caplacizumab rapidly corrects platelet count, decreases recurrence of TTP and decreases the rate of development of refractory disease in patients with TTP. However, it is associated with an increased risk of bleeding episodes, which are mainly mild to moderate.
